# Recognition of 7 genes signature (Cirrhosis Risk Score) in the diagnosed non-responders to DAAs therapy by intra-PBMCs nested HCV RNA PCR

**DOI:** 10.1186/s43141-023-00544-3

**Published:** 2023-08-30

**Authors:** Al-Shazly Gaber Mohamed Galal, Reham M. Dawood, Mostafa K. El Awady, Yasser Mohamed Mohamed El-Dessouky, Mohamed Mahmoud Abdel-Halim Mahmoud, Mohamed Darwish Ahmed Abd Alla

**Affiliations:** 1 Horus Specialized Hospital, General Authority of Health Care, Waburat Armant, Egypt; 2 Department of Microbial Biotechnology, National Research Center, Cairo, Egypt; 3 Department of Hepatology, Gastroenterology and Infectious Diseases, Faculty of Medicine, Al-Azhar University, Cairo, Egypt

**Keywords:** CRS, DAAs, PBMCs PCR, HCV Relapse, Liver fibro-cirrhosis

## Abstract

**Background and aims:**

Predictors of chronic HCV response to oral antiviral therapy (OAT) are related to host genetic variations. Single nucleotide polymorphisms (SNP) and alleles variations of host genes in association with hepatic fibro-cirrhotic changes have a distinct role in OAT outcomes. The current research evaluated the association of Cirrhosis-Risk-Scores (CRS) values, based on the correlation of seven genes signature-SNPs, with sonographic liver parenchymal changes in determining OAT outcomes.

**Methods:**

All study subjects (*n* = 54) were recruited three months after completing OAT and classified into three groups. Group I (*n* = 21) had negative HCV PCR, group II (*n* = 17) showed positive solitary intra-PBMCs HCV infection, and group III(*n* = 16) was serum HCV RNA PCR-positive. All study-population were subjected to examination by hepatic-ultrasound (US), FIB-4-scoring, and screening for 7 gene-signature that addressed CRS values as low, intermediate, and high depending on gene SNPs identification.

**Results:**

Group I showed a significant association with low CRS values compared to other groups (*P* < 0.001). Solitary intra- PBMCs HCV infection in group II was significantly combined with intermediate CRS values in comparison to groups I and III (*P* < 0.001). The high CRS values were significantly found in group III when compared to groups I and II (*P* < 0.01). On US imaging, low CRS values were common in normally appeared hepatic parenchyma (*P* < 0.001) and high CRS values were frequent in coarse-liver (*P* < 0.001), while bright-liver-tissues appearance was mainly detected in the intermediate CRS category (*P* = 0.09). On FIB-4 scoring, high CRS value were associated with hepatic fibro-cirrhosis compared to intermediate (*P* < 0.001) and low (*P* = 0.08) CRS-categories.

**Conclusion:**

The current study concluded the association of (a) high CRS values with coarse liver in viral-RNA serologic relapse, (b) low CRS values with normal liver tissues in sustained virologic response (SVR), (c) intermediate CRS values with bright liver in solitary PBMCs relapse.

## Introduction

There are conflicting reports regarding positive, negative, inconclusive, and denied association of certain gene SNPS and/or alleles with chronic HCV induced hepatic cirrhosis in different populations worldwide [1, 2]. On the other hand, direct-acting antivirals (DAAs) have markedly improved resistance to chronic HCV treatment and offered a unique chance to evaluate roles of host genetics that may participate in inducing hepatic fibro-cirrhotic changes as well as response to therapy [3, 4].

In chronic naive and post-treatment HCV RNA seropositive patients, evaluation of infection risk factors, as well as the correlation of infection courses with host immune-pathologic changes and treatment outcomes, are convenient tasks [5, 6]. On the other hand, diagnosing active chronic HCV infection in the RNA-seronegative population has been a challenge until developing cellular PCR that addressed intra-hepatocytes and intra-PBMCs RNA detection in both cryptogenic and occult viral infection [7, 8]. Grading of active hepatic tissues fibro-cirrhotic changes was addressed in chronic HCV infection in viremic patients and in solitary intracellular viral infections (who had negative serum viral RNA PCR) [6, 7]. Progressive fibro-cirrhotic liver tissues changes were found to be associated with solitary intra-bone marrow mononuclear cells (BMMCs) and intra-peripheral blood mononuclear cells (PBMCs) HCV infections with increasing hepatic-cirrhosis grades on the presence of HCV RNA viremia (positive serum PCR) [publication in progress].

The improving US resolution quality, the affordable overhead coast (reasonable machine price and the average screening time per patient), and the well-trained experienced operators’ availability make US screening more convenient and clinically applicable in both inpatient and outpatient clinics. Combining US image with Fibroscan in evaluation of hepatic parenchymal changes added more help in fibro-cirrhotic grading before and after treatment of chronic HCV infection. The results of both procedures (US and Fibroscan) in detecting and grading liver tissues changes are comparable to a considerable extent and enabled researchers to use one of them with satisfactory outcomes. The recognized absent hepatic tissue changes, bright liver parenchyma, and coarse hepatic surface by the US are respectively corresponding to normal (up to 5 kPa), up to moderate fibrosis (more than 5 but less than 15 kPa), and cirrhosis (≥ 15 kPa) by Fibroscan [9]. FIB-4 scoring is a biochemical-dependent scoring system that evaluates hepatic tissue changes and confirms the above-mentioned liver tissue grading by US [8, 10]. Sequelae of chronic HCV infection are variable among different hosts; they show no, slow, intermediate, and rapid progress in years or even decades to fibro-cirrhotic changes in liver tissues [11]. Several variables have been identified in cross-sectional and longitudinal studies that influence outcomes of chronic HCV infection. Environmental and host factors have major impacts on the progressive course of liver cirrhosis, while HCV genotype and blood loads of viral particles show minor effects [12, 13]. Host factors include male gender [14] and age of the subject at the time of infection [15], in addition to the presence of immunocompromising disorders and metabolic disease syndrome, particularly type II diabetes; all are associated with evident progressive liver disease [16, 17].

The available texts describe the association of liver tissue fibro-cirrhosis progression in chronic HCV infection with many single-nucleotide polymorphisms (SNP) in the genes encoding for IFN, TNF, interleukin-10, low-density lipoprotein, factor V Leiden, and the monocyte chemotactic protein 2 [13, 18–20]. In 2007, Huang et al. [21] addressed seven gene variant signatures known as cirrhosis risk scores (CRS) that are elaborated in association with hepatic cirrhosis in chronic HCV infection. High CRS scores that are derived from logarithms based on a constellation of seven SNPs showed a positive predictive value of 82% to 96% in diagnosing liver cirrhosis. A longitudinal study (follow-up for at least 60 months) was conducted to evaluate the prognostic value of the CRS seven-gene signature in chronically infected HCV patients who presented with no to minimal hepatic fibro-cirrhotic changes. The study concluded that host genetics, as defined by CRS, predicted hepatic fibrosis occurrence and progression in males with initially mild chronic HCV infection. The study expressed the probability of the CRS seven-gene signature to become a useful indicator for prognostic evaluation and may be for treatment decisions [22, 23]. Evaluation of host CRS seven gene signature in chronic HCV induced hepatic fibro-cirrhotic changes of SVR in addition to post-treatment serum and solitary cellular viral relapses is the target of the current research.

## Methods and subjects

### Patients

This study was carried out et al.-Azhar university hospitals on 54 patients who received HCV treatment (400 mg Sofosbuvir plus 60 mg Daclatasvir) with or without ribavirin (up to 1200 mg according to body weight). All patients were submitted to both serum and PBMCs HCV SRT-PCR 3 months after completing OAT. Patients were classified into three groups: (a) group I: 21 patients who had negative serum and PBMCs SRT-PRCs, (b) group II: 17 patients had positive solitary PBMCs-PCR, negative serum SRT-PCR, (c) group III: 16 patients presented with positive serum HCV-RNA and intra-PBMCs -RNA by SRT-PCR.

### Ethics

The authors have no conflicts of interest related to this research. All procedures performed in the current work that involved human participants were in accordance with the ethical standards of the institution and with the 1964 Helsinki Declaration and its later amendments or comparable ethical standards. Written informed consent was obtained from all individual participants included in the current study.

### Detection of HCV RNA in serum and PBMCs by SRT-PCR

#### Extraction of RNA from PBMCs

Peripheral blood (200 μL) was diluted with 10 mL of freshly prepared red blood cell alkaline buffer (38.8 mmol/L NH4Cl, 2.5 mmol/L K2HCO3, 1 mmol/L EDTA pH 8.0). After 10 min incubation at room temperature, the nucleated cells were washed with the same buffer and lysed in 500 mL anti-nuclease solution (4 mol/L guanidinium isothiocyanate containing 25 mmol/L sodium citrate, 0.5 sarcosyl and 0.1 mol/L ß-mercaptoethanol). A single step method previously described [24, 25] and subsequently modified [26] was fused to extract RNA.

#### Detection of both sense and antisense strands of HCV RNA

Briefly, the reaction mixture (50 μL) contained 400 ng of cellular RNA, RT-PCR bead (HVD), 50 pmol from each of the primers (1CH, P2, and 2CH) for amplification of the sense strand, or 50 pmol from P2 and 75 pmol from 2CH for antisense strand and 20 U of AMV reverse transcriptase. The mixture was incubated at 42 °C for 1 h and denatured for 15 min at 94 °C. Amplification of cDNA First-round PCR was performed in a thermal cycler for 30 cycles (94 °C for 1 min, 55 °C for 1 min, and 72 °C for 1 min). Nested PCR amplification was like the first-round PCR except for using 10 μL from the first PCR and two nested primers (P3 and P4) and 2 U Taq DNA polymerase. To assure the specificity of the assay two controls were employed: C1, ddH2O instead of RNA in the cDNA synthesis reaction to exclude RNA contamination; C2, PCR step with only F1 or R1 to exclude mixed primer contamination. In all conditions, the two controls provided negative amplification; special attention was paid to heat inactivating the reverse transcriptase at 95 °C for 1 h to reduce false detection of the antisense strand before adding the forward strand. Primer sequences used included below Primers used for PCR. Primer Sequences: 1CH *5-ggtgcacggtctacgagacctc-3*′; 2CH *5 aactcatgtcttcacgcagaa-3*; P2 *5-tgctcatggtgcacggtcta-3*′ P3; *5-ctttcgcgacccaacactac-3*′; P4 *5-agagccatagtggtctgcgg-3*’ [6, 26].

#### Correlation of ultrasonography (US) image with FIB-4 score

Chronic HCV induced parenchymal changes of the liver were examined by US image and were correlated with FIB-4 scoring system in all study populations. US images were classified into undetectable, bright, and coarse hepatic parenchyma. These three categories were correlated with the corresponding values of the FIB-4 score. The latter was calculated according to an equation that depends on liver function tests, platelets count and age of the patient: (Age X AST)/(platelet count) X [√ (square root) ALT]. The FIB-4 score ranges were segregated as follows: (a) low: < 1.45 corresponds to F0-F1, (b) intermediate: 1.45–3.25 equals to F2–F3, (c) high: > 3.25 is the same as F4 [10].

#### CRS genotyping (7 gene screening)

The 7 gene SNPs identified previously by Huang et al. [21] were genotyped using real-time PCR protocol based on the pre-validated TaqMan MGBTM probe for allele discrimination assay (Applied Bio systems). Briefly, 1.25 μL of a × 40 combined primer and probe mix (ABI/Life Technologies, USA) was added to 12.5 μL of 2 × TaqMan® Universal PCR master mix (ABI/Life Technologies, USA) in a 25-μL final volume of DNase/RNase free water (Invitrogen/ Life Technologies, USA) & template. The cycle conditions were 95 °C for 10 min, 95 °C for 15 s, and 60 °C for 1 min. The last two steps were repeated 40 times. The PCR run was performed on Rotor-Gene real-time PCR system (Qiagen, Santa Clarita, CA). Allelic discrimination plots were produced in Statistical Package for The Social Sciences.

Table 1 illustrates that each SNP can take the value 0 or 1 based on the obtained genotype, and then, each value has two probabilities (assuming that the patient can be cirrhotic or non-cirrhotic). Each SNP was calculated independently of other SNPs. The values obtained from Table 1 were substituted in the following Naive Bayes formula:

**Table 1:** CRS algorithm deduced by Huang et al.

Marker	Gene	SNP = 1	SNP = 0	P(SNP = 1l cirrhosis)	P (SNP = 1l no cirrhosis)	P(SNP = 0l cirrhosis)	P (SNP = 0l no cirrhosis)
SNP1	AZIN1 (Chr8)	GG	GA, AA	0.928030303	0.801282051	0.071969697	0.198717949
SNP2	TLR4 (Chr9)	CC	CT, TT	0.928301887	0.810126582	0.071698113	0.189873418
SNP3	TRPM5 (Chr11)	TT	TC, CC	0.318181818	0.487341772	0.681818182	0.512658228
SNP4	(AP3S2) (Chr15)	GG	GA,AA	0.554716981	0.696202532	0.445283019	0.303797468
SNP5	none (Chr1)	GG	GC, CC	0.78490566	0.610062893	0.21509434	0.389937107
SNP6	(STXBP5L)(Chr3)	GG	GA,AA	0.78490566	0.905660377	0.21509434	0.094339623
SNP7	AQP2 (Chr12)	GG	GC, CC	0.747169811	0.578616352	0.252830189	0.421383648

$${\left(\mathrm{CRS}\right)}=\frac{0.626\;\mathrm{ X\;p}\left(\mathrm{s}/\mathrm{ cirrhosis}\right)}{0.626\;\mathrm{ x\;p }\left(\frac{\mathrm{s}}{\mathrm{cirrhosis}}\right)+0.374\;\mathrm{ x\;p }(\frac{\mathrm{s}}{\mathrm{no}}\mathrm{cirrhosis})}$$

### Statistics

The collected data were analyzed using appropriate tests. Continuous variables were expressed in mean and standard deviation, while ordinal and nominal categorical data were described as number and percentage. The Statistical Program for Social Science (SPSS) version 18.0 was used to analyze the current data. A one-way analysis of variance (ANOVA) when comparing between more than two means. Chi-square (X2) test of significance was used to compare proportions between two qualitative parameters. *P* value < 0.05 was considered significant.

## Results

### Outcomes of DAAs therapy in relation to HCV-PCR results in the studied populations

Table 2 showed the outcomes of DAAs treatment (SOF + DAC versus SOF + DAC + RBA) in the studied population. There was a significant success of the SOF Plus DAC regimen without relapse in group I compared to groups II and III (*P* < 0.001). The difference in success rates on using the same regimen was insignificant on comparing group II with III (*P* = 0.42).

**Table 2:** Comparison of DAAs therapeutic regimens outcomes in all study population

*HCV PCR results*	SOF + DAC (*n* = 39)	SOF + DAC + RBA (*n* = 15)
A. Group I: − ve HCV PCR (*n* = 21)	21/21(100%)	0(0%)
B. Group II: + ve cellular/ − ve serum PCR (*n* = 17)	9/17(52.94%)	8/17(47.06)
C. Group III: + ve serum HCV PCR (*n* = 16)	9/16(56.25%)	7/16(45.75%)
*P* value: A vs B	**0.002**	
: A vs C	**0.005**	
: B vs C	0.42	

SOF plus DAC regimen had a highly significant efficiency in group I when compared to groups II and III (*P* < 0.001), while comparing the outcomes of the same regimen in group II with group III showed an insignificant difference (*P* = 0.429)

*Abbreviations*: *DAAs* Direct-acting antivirals, *SOF* Sofosbuvir, *DAC* Daclatasvir, *RBA* Ribavirin, − *ve* negative, + *ve* positive, *vs* versus. Mid-P Exact 1-tailed *P* was used to analyze data

### Grading of cirrhosis risk score (CRS) in relation to HCV-PCR results after DAAs therapy in all study groups

The cirrhosis risk score (CRS) grading results were demonstrated in Table 3. The CRS had significant frequencies of low grades (< 0.5) in post-treatment negative HCV-PCR populations (group I) in comparison to cases with either solitary positive cellular viral-RNA PCR (group II) or positive serum PCR (group III) (*P* < 0.001). On the other hand, the intermediate CRS values (ranging from 0.5 to 0.7) showed significant association with solitary HCV intracellular infection (group II) compared to positive serum viral PCR group III (*P* < 0.005), but insignificant changes in comparison with infection-free group I (*P* = 0.36). Viremic patients with positive HCV-RNA serology in group III showed a significant association with high CRS values (> 0.7) when compared with either SVR group I (*P* < 0.001) or solitary intracellular HCV infection in group II (*P* < 0.001). At the same time, solitary intracellular HCV infection showed a significant association with high CRS values in comparison with infection-free subjects in group I (*P* < 0.001).

**Table 3:** Comparison of CRS score values (low, intermediate, and high) among the three studied groups

*The studied groups*	CRS score to assess risks of liver tissues damage		
	Low score range (*n* = 11)(< 0.5)	Intermediate score (*n* = 19) (range from 0.5 to 0.7)	High score (*n* = 24) (> 0.7)
A. Group I: − ve HCV PCR (*n* = 21)	11/21(52.38%)	10/21(47.62%)	0/21(0%)
B. Group II: + ve cellular/ − ve serum PCR (*n* = 17)	0/17(0%)	8/17(47.06%)	9/17(52.94%)
C. Group III: + ve serum HCV PCR (*n* = 16)	0/16(0%)	1/16(6.25%)	15/16(93.75%)
*P* value: A vs B	0.001	0.36	0.005
: A vs C	0.002	0.003	< 0.001
: B vs C	NA	0.005	0.005

HCV-infection-free group I showed a significant association with low CRS values compared to groups II and III (*P* < 0.001). Solitary intracellular viral RNA infection in group II was significantly associated with intermediate CRS values in comparison to both SVR subjects in group I and serologic RNA relapse in group III (*P* < 0.001). The high CRS values were found more often in HCV RNA seropositive group III when compared with virally infection-free group I and solitary intra-cellular virally infected group II (*P* < 0.01). *Abbreviations*: *DAAs* Direct-acting antivirals; − ve, negative; + ve, positive; vs, versus. Mid-P Exact 1-tailed *P* was used to analyze data

### Association of various CRS values with hepatic parenchymal changes as defined by FIB-4 score

As illustrated in Table 4, the intermediate values of CRS were frequently associated with normal ranges of FIB-4 scores compared to both low (*P* = 0.06) and high CRS values (*P* = 0.005). On the other hand, high CRS values were seen more often in hepatic fibro-cirrhotic changes compared to normal (*P* = 0.08) and intermediate (*P* < 0.001) CRS values.

**Table 4:** Correlation of cirrhosis risk score (CRS) values with grades of liver tissue changes by FIB-4-Scores

CRS score	FIB-4-Score in liver tissues changes assessment	
	Within the normal range (up to 1.45) (*n* = 33)	Fibro-cirrhotic changes (> 1.45) (*n* = 21)
A. Low (*n* = 11)	7/11(63.64%)	4/11(36.36)
B. Inter. (*n* = 19)	17/19(89.47%)	2/19(10.53%)
C. High (*n* = 24)	9/24(37.5%)	15/24(62.5%)
*P* value: A vs B	0.06	
: A vs C	0.08	
: B vs C	0.005	

There was a highly significant association of high CRS values with FIB-4 hepatic fibro-cirrhotic changes (*P* < 0.001), with an insignificant difference in the association of low and intermediate CRS with a normal range of the same score (*P* > 0.05). Mid-P Exact 1-tailed *P* was used to analyze data

### Correlation of CRS values with liver parenchymal fibro-cirrhotic changes as recognized by ultrasound (US)

Table 5 addressed the association of low, intermediate, and high CRS values with normal, fibrotic, and cirrhotic liver parenchyma defined by the US. Over 90% of low CRS values were present in populations with normal US images; *P* = 0.06 and < 0.001 on comparison with intermediate and high CRS values respectively. There was also a significant association of intermediate CRS values with normal liver image by the US compared to the same association with high CRS values *P* < 0.001). The early hepatic fibro-cirrhotic changes (bright liver by the US) were equally distributed within the three categories of CRS values (*P* > 0.05). On the other hand, high CRS values were significantly associated with liver cirrhosis by the US in comparison with either low or intermediate CRS values (*P* < 0.001).

**Table 5:** Association of CRS score values with grades of hepatic parenchymal changes by US imaging in all groups

CRS score	Ultrasound (US) images in liver tissues changes assessment		
	Normal US imaging (*n* = 22)	Bright by US imaging (*n* = 12)	Coarse surface by US imaging (*n* = 20)
A. Low (*n* = 11)	10/11(90.91%)	1/11(9.09%)	0(0%)
B. Inter. (*n* = 19)	12/19(63.16%)	6/19(31.58%)	1/19(5.26%)
C. High (*n* = 24)	0/24(0%)	5/24(20.83%%)	19/24(79.17%)
*P* value: A vs B	0.06	0.09	0.31
: A vs C	< 0.001	0.22	< 0.001
: B vs C	< 0.001	0.22	< 0.001

Low CRS values were associated with normal US hepatic image (*P* < 0.0001), while high CRS values were more frequent in the cirrhotic liver (*P* < 0.001). Early hepatic fibrosis (bright liver) was equally distributed in the three CRS categories (*P* > 0.05). Mid-P Exact 1-tailed *P* was used to analyze data

## Discussion

The studied population in the current research are recruited three months after completing oral antiviral therapy (OAT) for 12 weeks. All study populations are tested by both serum and PBMCs HCV-PCR as well as screening by both hepatic US and FIB-4 scoring system. They are segregated into subjects with sustained virologic response (SVR) (group I), solitary intracellular HCV-RNA infection with negative serum viral PCR (group II), and patients with positive serum HCV-PCR (group III). Association of oral antiviral therapy (OAT) outcomes and seven gene signatures (SNPs dependent) presented as cirrhosis risk score (CRS) values with different grades of hepatic parenchymal changes is tested in the currently studied small numbers of populations as a pilot study. CRS scores are categorized into low (< 0.5), intermediate (from 0.5 to 0.7), and high (> 0.7) values. The association of each CRS category with normal, bright, and coarse liver parenchyma in SVR, solitary intracellular HCV infection and viral RNA seropositive patients is addressed by the illustrated research above.

Outcomes of OAT in association with specific host genes variants results in chronic HCV infection are still debatable because of variation in the diagnostic procedures used to address SVR, probabilities of viral relapses, and differentiation of resistance to OAT from actual HCV-RNA serologic relapse [8, 27, 28]. Post-treatment persistence of intracellular HCV infection is considered viral relapse and/or resistance to antiviral therapy as it is followed later by viral RNA seroconversion [6, 29–31]. Different patterns of SNPs distribution are identified when specific human genes are sequenced, which effectively play distinct roles in determining treatment outcomes that have been based solely on screening for serum HCV-PCR [1, 4, 21, 22]. However, pending cases of serologic HCV relapse are expected when negative serum HCV-PCR is associated with the positive intracellular viral infection. Genotyping of these cases (pending HCV serologic relapse) to address SNPs during host genes sequencings are targeted in the current research. Correlating host genes typing results of the pending relapse populations with hepatic fibro-cirrhosis grades showed the dominance of persistent solitary intracellular HCV infection in intermediate CRS values. The stationary status of bright hepatic parenchyma with a lacking ability to progress into cirrhosis or regress back to normal liver tissues is explained by not only the relationship to duration of infection but also to variations in host genetic constituent. So, the bias of a hidden intracellular viral infection that would end up with both unexpected serologic relapse and upgrading of hepatic fibro-cirrhotic changes [6, 29–31] can be minimized by the appropriate management of solitary intra-cellular HCV infection.

In addition, the broad-spectrum use of ultra-sonographic (US) images worldwide in intra-abdominal solid organs evaluation is attributed to the improving high-quality resolution because of advanced technology, affordable cost on comparing reasonable machine price with the average screening time per patient, and availability of the well-trained experienced operators. These advantages make the US screening procedure one of the most convenient and attractive methods to implement everywhere for liver tissue screening before and after OAT. Adding scanning of hepatic parenchyma by fibroscan to the US image has refined the differentiation of cirrhosis from fatty infiltration in addition to fine grading of both conditions. Fortunately, results from both imaging procedures (US and Fibroscan) in detecting and grading hepatic parenchymal changes are conclusively comparable to the degree that enable researchers to use them either complimentary or singular with dependable satisfactory outcomes. The reported no hepatic tissue changes, bright hepatic parenchyma, and coarse liver surface by US are respectively corresponding to normal [up to 5 kPa], mild to moderate fibrosis (> 5 but < 15 kPa), and cirrhosis (15 kPa or above) by Fibroscan [9]. The FIB-4 scoring system is a biochemical dependent evaluation of hepatic parenchymal changes that is usually used to confirm the above-mentioned liver tissue changes by the US [10]. Accordingly, the use of US in the evaluation of hepatic parenchymal changes in the current research is justified upfront in literature by data from Fibroscan grading and currently by the results from the FIB-4 scoring system. Therefore, the convenience of affordability and reliability of the US image is much appreciated at baseline and follow-up evaluation of hepatic parenchymal changes on the application of well-defined interventions that require correlation with various host-related immunogenetic factors.

The Association of CRS changes in chronic HCV infection with hepatic parenchymal fibro-cirrhotic changes is demonstrated in several studies [21, 32]. Moreover, the high CRS score [33]. The current research studies the relationship between various CRS values (low, intermediate, and high) and outcomes of OAT in chronic HCV infection. Fortunately, in this study, OAT outcomes are defined as SVR when both serum and cellular HCV PCR are negative, while viral relapses are diagnosed when viral RNA and/or its remnant are found in either serum and/or PBMCs [6, 29–31]. So, probabilities of the unpredictable infectious agents such as hidden intracellular viral genomic materials (pending viral relapse) to bias the currently elaborated data and their subsequent conclusions are wearing thin. It is obvious in the current data set that proving hepatic tissue fibro-cirrhotic changes by liver US and/or FIB-4 score calculation is crucial to interpret the association of various levels of CRS values with (a) complete elimination of HCV particles from serum and cells, (b) persistent intracellular viral infection without serologic relapse, (c) fully blown HCV serologic relapse. It is currently reported that (1) SVR, as indicated by viral infection-free serum and cells, are associated with low values of CRS (< 0.5) when liver tissues appear normal by US. (2) Serologic relapse with positive serum HCV-PCR is associated with high CRS-values (> 0.7) when live tissues are cirrhotic (coarse by US). Both findings are in full agreement with the previous studies [21, 34]. The association of intermediate values of CRS with solitary intracellular HCV-RNA detection in the early fibrotic liver (bright appearance by the US) is a novel finding that is supported by IL28B gene sequencing in previous studies from our lab [35]. The interpretation of this finding may be related to variations in the genetic construction of the host that plays a significant role in HCV infection outcomes. However, clearance of post-OAT intracellular HCV remaining antigenic positive or replicative negative strands by the continuation of OAT therapy for a longer duration than the anticipated time in the primary therapeutic protocol [36] may indicate the iatrogenic origin of this phenomenon. Extension of this study should be performed on larger sample size to prove this finding.

## Conclusion

The current study addressed the following: (a) high CRS values are present in liver cirrhosis (coarse liver by the US) when serum HCV-PCR is positive, (b) low CRS values are found in normally appeared liver tissues by the US when both serum and cellular HCV PCR are negative, (c) Intermediate CRS values are recognized more often in early fibrotic changes (bright liver by the US) in association with solitary intra-PBMCs HCV-RNA infection. So, we recommend correlating cirrhosis risk score (CRS) values with ultrasonographic patterns of hepatic parenchyma before oral antiviral therapy (OAT) as predictors for treatment outcomes in chronic HCV infection.

## Data Availability

All data generated or analyzed during this study are included in this published article.

## Abbreviations

CHIKV: Chikungunya virus
OAT: Oral antiviral therapy
CRS: Cirrhosis Risk Score
HCV: Hepatitis C virus
DAAs: Direct-acting antivirals
US: Ultrasound
PBMCs: Peripheral blood mononuclear cells
FIB-4: Fibrosis scoring system
IFN: Interferon
TNF: Tumor necrosis factor
SVR: Sustained-virologic-response
PCR: Polymerase chain reaction
SNPs: Single-nucleotide polymorphisms
